# Anti-EGFR-Targeted Therapy for Esophageal and Gastric Cancers: An Evolving Concept

**DOI:** 10.1155/2009/804108

**Published:** 2009-07-14

**Authors:** Tomislav Dragovich, Christopher Campen

**Affiliations:** ^1^Arizona Cancer Center, 1515 N. Campbell Avenue, Room 1969G, P.O. Box 245024, Tucson, AZ 85724, USA; ^2^Department of Pharmacy Practice, College of Pharmacy, University of Arizona, P.O. Box 210202, Tucson, AZ 85724, USA

## Abstract

Cancers of the esophagus and stomach present a major health burden worldwide. In the past 30 years we have witnessed some interesting shifts in terms of epidemiology of esophago gastric cancers. Regardless of a world region, the majority of patients diagnosed with esophageal or gastric cancers die from progression or recurrence of their disease. While there are many active cytotoxic agents for esophageal and stomach cancers, their impact on the disease course has been modest at best. Median survival for patients with advanced gastroesophageal cancer is still less than a year. Therefore, novel strategies, based on our understanding of biology and genetics, are desperately needed. Epidermal growth factor receptor (EGFR) pathway has been implicated in pathophysiology of many epithelial malignancies, including esophageal and stomach cancers. EGFR inhibitors, small molecule tyrosine kinase inhibitors and monoclonal antibodies, have been explored in patients with esophageal and gastric cancers. It appears that tumors of the distal esophagus and gastroesophageal junction (GEJ) may be more sensitive to EGFR blockade than distal gastric adenocarcinomas. Investigations looking into potential molecular predictors of sensitivity to EGFR inhibitors for patients with esophageal and GEJ cancers are ongoing. While we are still searching for those predictors, it is clear that they will be different from ones identified in lung and colorectal cancers. Further development of EGFR inhibitors for esophageal and GEJ cancers should be driven by better understanding of EGFR pathway disregulation that drives cancer progression in a sensitive patient population.

## 1. Introduction

The estimated incidence of esophageal and gastric cancer in the United States is 16 470 and 21 500 in the United States, respectively, in 2008 [1]. Worldwide figures indicate nearly 1 300 000 new cases and an overall mortality of approximately 1 100 000 patients between esophageal and gastric cancers [2], which underscores the global challenge in dealing with these diseases. East Asia makes up for a significant proportion of new cases, with very high rates of gastric and esophageal cancer in China and Japan [3]. Some of the risk factors for the development of esophageal or gastric cancer overlap, including nutritional factors such as smoking and alcohol use. There is however a tremendous heterogeneity in terms of epidemiology of esophageal and gastric cancer. While in developing countries proximal squamous cell esophageal cancers and gastric cancers with intestinal or diffuse type histology still predominate, we have witnessed an epidemiological shift in developed countries, including the United States [4]. This relates not only to tumor histology, with esophageal adenocarcinoma now surpassing squamous carcinoma in incidence, but also to changes in primary tumor location. Adenocarcinomas of the distal esophagus and gastroesophageal junction are becoming increasingly more common than distal gastric cancers in the US and Western world. Interestingly, we are beginning to see the beginning of this trend in some countries in Latin America and Asia in the last decade. The causes of this epidemiological shift are still unclear although there is a suggestion that this phenomenon may be, at least in part, related to eradication of *Helicobacter pylori* infection in developed countries and increased incidence of gastroesophageal reflux disease in Western world. Significant and recurrent gastroesophageal reflux disease (GERD) is associated with an eightfold increased risk of developing adenocarcinoma of the esophagus [5]. Approximately 5 to 8 percent of patients with GERD develop Barrett's esophagus, a disease characterized by dysplasia of the normal epithelium [6]. Patients with Barrett's are at a high risk of development of adenocarcinoma of the esophagus along with the gastroesophageal junction (GEJ). *Helicobacter pylori* (*H. pylori*) infection, on the other hand, has been shown to be a significant risk factor for the development of distal gastric cancer [7].

The development of targeted therapies for the treatment of cancers has really taken off recently, with 17 targeted therapies approved by the Food and Drug Administration (FDA) since 2000 [12]. The novel targeted therapies include monoclonal antibodies or small molecule inhibitors targeting either growth factors or growth factor receptor kinases. Of these agents, epidermal growth factor receptor (EGFR) inhibitors have played a visible role in the management of solid malignancies including colorectal cancer, metastatic non small-cell lung cancer (NSCLC), pancreatic cancer, and squamous-cell carcinoma of the head and neck (HNSCC). Currently, there are four EGFR inhibitors approved by the FDA including two small molecule tyrosine kinase inhibitors (erlotinib and gefitinib) and two monoclonal antibodies (cetuximab and panitumumab) [13–17]. The clinical use of EGFR inhibitors will likely continue to increase in the future for two main reasons. First, there are many EGFR inhibitors in the later stages of development [18]. Second, new indications for the current and novel agents are being actively pursued. This review article focuses on current experience in using therapeutic EGFR inhibitors as a therapy for patients with esophageal and gastric cancers.

## 2. EGFR Pathway and Implications for Therapy of Gastroesophageal Cancers

EGFR, or ErbB1, is a transmembrane receptor and a member of four structurally related tyrosine kinases. EGFR is composed of an extracellular binding domain, a transmembrane portion, and an intracellular cytoplasmic domain with tyrosine kinase functionality. In the event of ligand binding, either homodimerization or heterodimerization can occur. This process leads to tyrosine kinase autophosphorylation and activation [18]. Downstream of EGFR dimerization and activation are multiple processes that can result in cancer cell proliferation, prevention of apoptosis, tumor-induced angiogenesis, and activation of invasion and metastatic growth [19, 20].

The available therapeutic EGFR inhibitors include two classes of agents, monoclonal antibodies and small molecule tyrosine kinase inhibitors. There are significant pharmacological and therapeutic differences between the two classes of agents, which are clinically important. Small molecule tyrosine kinase inhibitors can bind intracellularly at the tyrosine kinase binding domain through competition with ATP. In contrast, monoclonal antibodies bind extracellularly, blocking ligand binding and dimerization of the receptor. Some monoclonal antibodies may have an additional mechanism of action through immune system activation. Immune system activation can result in antibody-dependent cellular cytotoxicity (ADCC) and activation of the complement system [25]. Another significant difference between monoclonal antibodies and small molecule inhibitors is the specificity of the agent. Monoclonal antibodies are very selective in nature, while small molecule TKIs can inhibit additional kinase receptors. This can theoretically increase the efficacy, but may have deleterious effect on the side effect profile [17]. Additionally, there are noteworthy pharmacokinetic differences between the two classes. Small molecule tyrosine kinase inhibitors such as erlotinib and gefitinib are dosed orally on a continuous daily basis due to short half lives. In addition, oral administration may not be practical or effective for some patients with gastrointestinal malignancies due to the lack of anatomic integrity or decreased absorption caused by primary malignancy. Monoclonal antibodies such as cetuximab and panitumumab can only be given intravenously, but have an extended half life of approximately seven days [26]. This does offer increased adaptability of the antibody dosing in regard to a specific regimen (weekly or biweekly), and future studies are exploring feasibility of further prolonging dosing intervals [18, 27]. The disposition of monoclonal antibodies is also more straightforward, as they are cleared and recycled by reticuloendothelial cells, mostly in the liver. This is in contrast to small molecule tyrosine kinase inhibitors metabolized by CYP450 system, which does create a possibility for potentially adverse interactions with other drugs and food ingredients.

An increasingly explored method of predicting the efficacy of EGFR inhibitors is through assessing the cutaneous adverse effects as a correlate of response. Rash is a common adverse effect of EGFR inhibitors and occurs in approximately 45%–100% of patients. Mechanistically, the rash is likely due to the expression of EGFR in the epidermal layers of the skin and is dose dependent [28]. Many studies have shown a consistent relationship between rash and both response to therapy and survival. The first study to report this finding was in patients with colorectal cancer [29] but has been also shown in patients with NSCLC [30], HNSCC [31], and ovarian carcinoma [32]. The observation has been seen with both small molecule TKIs and monoclonal antibodies targeting EGFR. As the rash is both dose dependent and correlates with survival, there is interest in increasing the dose in patients that do not develop a significant rash. In the EVEREST study, a phase I/II study of cetuximab in patients with metastatic colorectal cancer, patients were dose escalated until a greater than grade 2 adverse effect occurred or until a maximum dose of 500 mg/m^2^ [33]. Over half of the patients were able to achieve the maximum dose while on treatment. While the primary endpoint was not efficacy and the sample size was small, the single agent response rate was 30% in the escalating dose arm versus 13% in the standard dose arm. While the quality of life and discontinuation rates need to be considered when using this strategy, these results are promising and should be considered in future studies with EGFR inhibitors. 

## 3. Standard Chemotherapy for Esophageal and Gastric Adenocarcinomas

The establishment of standard chemotherapy for esophageal and gastric cancer still remains a moving target, despite of decades of intense clinical investigations [3]. While both esophageal and gastric cancers respond to many different cytotoxic agents, responses are usually short lasting and systemic chemotherapy so far have shown only a modest success in prolonging survival of patients with advanced or metastatic disease [34]. Five-year survival rate for esophageal cancer, all stages included, is only about 15%–25%. This underscores late diagnosis and limited efficacy of potentially curative modalities such as surgery and chemoradiation. For patients with unresectable or metastatic disease, which account for more of the 50% of new cases, prognosis is dismal, with a median survival of less than one year. The role of systemic therapy is palliation. Commonly used chemotherapy regimens for metastatic disease usually include a combination of fluoropyrimidine (5-fluorouracil or capecitabine) and a platinum drug (cisplatin, oxaliplatin or carboplatin) [35, 36]. Taxanes such as docetaxel and paclitaxel have activity, either alone or in combination with a fluoropyrimidine or platinum [37, 38]. Another promising, and also well-tolerated combination is a combination of irinotecan and cisplatin [39]. Older agents, such as anthracyclines (doxorubicin, epirubicin), and topoisomerase II inhibitors (etoposide), or vinca (navelbine), have also been used with a modest success [40]. While there is evidence of some incremental improvement with regard to efficacy and tolerability of chemotherapy combinations, their impact on the natural history of esophageal cancer has been disappointing thus far [41].

For gastric adenocarcinomas, a commonly used regimen is a combination of 5-fluorouracil and cisplatin (CF). More recently, combination regimens such as ECF (epirubicin, cisplatin and fluorouracil) and DCF (docetaxel, cisplatin and fluorouracil) have demonstrated increased efficacy compared to CF but at the expense of additional toxicity [42–44]. Substitution of 5-fluorouracil with capecitabine and of cisplatin with oxaliplatin has resulted in encouraging activity and good tolerability (REAL trial) [45]. Continuous infusion is better tolerated than bolus 5-fluorouracil, especially when it is combined with other drugs, such as irinotecan or oxaliplatin in patients with gastroesophageal carcinomas [46, 47]. S-1 (TS-1) is another oral fluoropyrimidine that has been approved for the therapy of gastric cancer in Japan; confirmatory trials are in progress in Europe and the US [48]. Despite addition of several novel cytotoxic drugs, the median survival for patients with locally advanced unresectable or metastatic gastric cancer still falls short of reaching 12 months. Thus, there is a real need to expand therapeutic options for this group of patients. Lately the focus has been on targeted therapeutics. Newer and better tolerated combination regimens also provide a superior platform for adding and testing novel targeted agents.

## 4. Evidence for EGFR Pathway Disregulation in Gastric and Esophageal Malignancies

The importance of the EGFR receptor lies in the downstream effects of activation. The primary intracellular pathways implicated following phosphorylation of EGFR are the phosphoinositol-3-kinase (PI3K)/Akt and RAS/mitogen-activated protein kinase (MAPK) pathways [49, 50]. The PI3K pathway is involved in apoptotic and survival signaling, and downstream of this pathway is the mammalian target of rapamycin (MTOR). The RAS/MAPK pathway is involved in cancer cell proliferation, which is responsible for progression from the G1 to S phase, and gene transcription [51].

In esophageal cancer, overexpression of EGFR by immunohistochemistry (IHC) is very common, occurring in approximately 80% of patients with adenocarcinoma and squamous cell carcinoma [52]. Additionally, amplification of the EGFR gene has been detected in some esophageal adenocarcinomas. Fluorescence in situ hybridization (FISH) analysis shows amplification in about 8%–30% of cases [53–55]. Multiple studies have shown that increased EGFR expression is associated with an overall decrease in survival in patients with esophageal cancer [56]. In contrast, overexpression of EGFR by IHC occurs less frequently in gastric cancer, at a rate of less than 40%. Additionally, in a large study of 511 patients only 2.3% of patients had gene amplification measured by FISH [57]. In this study overexpression of EGFR resulted in a statistically significant decrease in survival. Based on these findings, multiple phase I/II studies of small molecule tyrosine kinase inhibitors and monoclonal antibodies have been initiated for patients with esophageal and gastric cancers.

## 5. Clinical Trials of EGFR Inhibitors in Esophageal and Gastric Cancer

### 5.1. Tyrosine Kinase Inhibitors

Some of the first clinical trials of EGFR inhibitors in esophageal and gastric cancers were those involving small molecule tyrosine kinase inhibitors. Gefitinib (Iressa) was the first in the new class of small molecule inhibitors to be tested clinically. At doses of 250–500 mg/day gefitinib had demonstrated clinical activity, especially in chemotherapy refractory patients with non small cell cancer. Ferry et al. conducted a phase II trial in patients with advanced esophageal carcinoma [8]. Twenty seven patients with unresectable or metastatic adenocarcinoma of the esophagus or gastroesophageal junction, and no more then one prior chemotherapy regimen were treated with 500 mg/d of gefitinib. Overall the therapy was well tolerated with diarrhea and skin rash being the most common adverse events, as expected. The median overall survival was 4.5 months and progression free survival was 1.9 months. There were three (11%) partial responders and 26% of patients had stable disease as their best response. Two of the seven patients tested had EGFR mutations but were not predictive of response. The other markers of EGFR pathway activation were analyzed in paired biopsies but did not correlate with response. Again, due to small number of tissue samples analyzed correlative analyses were of the limited scope. In another study with gefitinib for esophageal cancer, authors tested pre- and post treatment tumor samples in 24 patients. However, no correlation of change in expression of EGFR, pAKT, and pERK was demonstrated [9]. Rojo et al. reported on a pharmacodynamic investigations of tumor biopsies obtained from patients with gastric (77%) and gastroesophageal junction (21%) carcinomas treated with two different doses of gefitinib 250 and 500 mg/d [58]. Authors were able to obtain 46 (out of 70 subjects) paired pre and post-treatment biopsies. Sample analysis was stratified as Japanese and non-Japanese patients and as lower and higher dose of gefitinib. Gefitinib therapy was associated with significant downregulation of phosphorylated EGFR, but not of pMAPK and pAKT. Interestingly, increase in apoptosis was associated with increased exposure (dose) to gefitinib. Although there was some evidence of biological effect on EGFR pathway, it did not translate in clinical benefit in this study. Of note, compared to the other two trials the majority of patients in this trial had distal gastric tumors (see Table 1).

The largest trial in this population was Southwest Oncology Group Trial 0127, which included 70 patients [10]. The patients were stratified on the basis of tumor location on (1) distal esophageal and gastroesophageal junction adenocarcinoma and (2) distal gastric adenocarcinomas. The gastric strata was closed after the first phase due to lack of activity (*n* = 26) while esophageal/GEJ strata completed full accrual (*n* = 46). Interestingly, all of the objective responses (1 complete and 4 partial) were observed in esophageal/GEJ arm (overall response rate 9%, CI 3–22%). Diagnostic archived biopsies were obtained on 54 patients and analyzed for EGFR, pAKT, and TGF-alpha by immunohistochemistry. There was no correlation with anti-tumor activity. Investigators also analyzed tumor biopsies for EGFR gene amplification and for mutations involving exons 18, 19, and 21. There was no evidence of EGFR gene amplification or any of selected mutations in 54 tested tissue specimens. In a separate study [59] authors investigated the stability of pAKT in specimens obtained by en-block resection versus those obtained by needle or endoscopic biopsies. There was great variability between two approaches, raising the concern about stability of phosphorylated kinases when tumor samples are obtained by different procedures and from different resources, in a setting of a multicenter trial.

Lapatinib, an oral inhibitor of EGFR and HER 2 was also tested in patients with upper gastrointestinal malignancies [11]. No objective responses were observed and only two of twenty five treated patients achieved disease stabilization.

### 5.2. Therapeutic Monoclonal Antibodies

Experience with anti-EGFR monoclonal antibodies is less extensive. Investigators from SWOG reported results of a phase II study of cetuximab (Erbitux) in 55 patients with metastaic esophageal adenocarcinoma [21] (see Table 2). The patients were allowed to have one prior chemotherapy regimen for advanced disease. The median overall survival was 4 months and there were three unconfirmed partial responses. A group from Memorial Sloan Kettering reported on their study of a combination of cetuximab plus irinotecan and cisplatin in irinotecan/cisplatin refractory patients with esophageal cancer [22]. Only one partial response was seen out of eight patients that were evaluable for response.

Two trials have been published on the use of cetuximab combination therapy for advanced gastric cancer patients. In a cetuximab + FOLFIRI trial involving 38 patients, 34 had untreated gastric adenocarcinoma [23]. Combination therapy results were promising with a median time-to-progression of 8 months. Correlative analysis of this study showed no association between either EGFR expression or rash and response to cetuximab. The combination of FOLFOX6 and cetuximab was also studied in 38 gastric cancer patients [24]. Response rates were similar to the previous trial at approximately 50%, but median time-to-progression was 5.5 months. Again, as in the previous trial, EGFR expression was not predictive of response to therapy or overall survival. Low levels of epidermal growth factor (EGF) and transforming growth factor alpha (TGF-a) did correlate with response, but had no statistically significant effect on overall survival.

Based on the currently available clinical data it appears that small molecule EGFR tyrosine kinase inhibitors have activity in gastroesophageal cancers. Trials with gefitinib and erlotinib have consistently demonstrated that the benefit is limited to about 10% of patients with distal esophageal and gastroesophageal junction carcinomas. Gastric adenocarcinomas appear to be resistant, at least in a mono therapy setting. This magnitude of anti tumor activity was seen with EGFR inhibitors in non small cell cancer and colorectal cancer and also with anti-HER2 therapy in patients with breast cancer. However, unlike with lung cancer (EGFR gene amplification, EGFR gene mutation, lack of KRAS mutation) and colorectal cancers (lack of KRAS mutations), molecular markers of sensitivity to EGFR blockade are currently unknown for gastroesophageal carcinomas. Despite a valiant effort to identify these markers, more robust and comprehensive tissue-based analyses are needed in order to better select patients with gastroesophageal adenocarcinomas that may derive clinical benefit from EGFR inhibitors.

## 6. Conclusion and Future Prospects

EGFR inhibitors have shown modest clinical activity, primarily in patients with esophageal and gastroesophageal junction adenocarcinomas. While there is an always present motivation to quickly integrate targeted therapies and combine them with cytotoxic drugs we believe that it is prudent to make some additional efforts in order to optimize efficacy of these agents before we launch in to large and expensive randomized trials. Could a subset of patients likely to benefit be prospectively identified on the basis of tumor genotype or pharmacogenomic testing? As we have seen, molecular drivers that determine sensitivity to EGFR inhibitors in esophageal and GEJ adenocarcinomas are different from those important in lung and colorectal cancers. This needs further investigation in order to be able to identify subset of patients that will benefit from EGFR blockade. Is it possible to further optimize efficacy by increasing dose of EGFR inhibitors in selected patients (i.e., treat to > grade 2 skin rash), which appears to be true for patients with colorectal cancer? As we have seen, adenocarcinomas of esophagus, gastroesophageal junction, and distal stomach are recognized as distinctive entities in terms of their pathophysiology and epidemiology. This is also likely to be true when we are considering biology of these tumors. By lumping together all these cancers in our clinical trials we are increasing the chance of diluting any significant clinical benefit and reducing our ability to make further progress in terms of drug development. Therefore, it is important that future trials in addition to histology stratify patients based on the location of their primary tumor (i.e., esophageal adenocarcinomas, gastroesophageal junction, and distal gastric tumors) and their molecular characteristics. We expect further advancement of this therapeutic concept for patients with esophago gastric cancers to be driven by development of novel and more potent EGFR inhibitors, along with the development of “omics” technology allowing for a more comprehensive pathway analysis, validation of biologic targets of interest and identification of specific biomarkers.

## Figures and Tables

**Figure 1:**
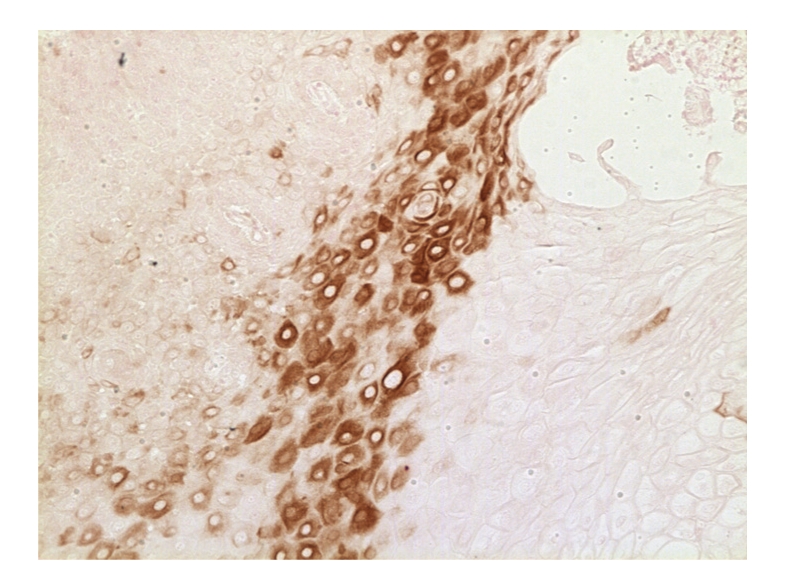
A section of human gastroesophageal adenocarcinoma stained by an anti-EGFR monoclonal antibody and biotin streptavidin 3,3′-diaminobenzidine method (Courtesy of Dr. Amanda Baker, University of Arizona).

**Table 1:** Trials of oral EGFR tyrosine kinase inhibitors.

	Phase	Number of patients	Anatomic site	Histology	Treatment regimen	Outcomes	Comments
Ferry et al. [8]	II	27	Esophagus	27/27 adenocarcinoma	Gefitinib 500 mg PO daily	mOS 4.5 months mPFS 1.9 months 3/27 PR (11%) 7/27 SD (26%)	Prior chemotherapy: 18/27 (67%)
Janmaat et al. [9]	II	36	Esophagus	26/36 adenocarcinoma (72%) 9/36 squamous cell (25%) 1/36 adenosquamous (3%)	Gefitinib 500 mg PO daily	mOS 5.5 months mPFS 2 months 1/36 PR (3%) 10/36 SD (28%)	Second-line treatment 8/36 not assessable for response
Dragovich et al. [10]	II	70	26/70 Gastric (37%) 44/70 GEJ (63%)	70/70 adenocarcinoma	Erlotinib 150 mg PO daily	mOS GEJ 6.7 months mOS Gastric 3.5 months mTTF GEJ 2 months mTTF Gastric 1.6 months GEJ: 1/43 CR (2%), 3/43 PR (7%), 5/43 SD (12%)	All responses in esophageal GEJ cohort No responses seen in the gastric cohort
Hecht et al. [11]	II	25	13/25 GEJ (52%) 12/25 Esophagus (48%)	25/25 adenocarcinoma	Lapatinib 1000–1500 mg PO daily	No responses seen 2/25 SD (8%)	Elevated TGF-alpha expression correlated with shorter TTP

**Table 2:** Trials of anti EGFR monoclonal antibodies.

	Phase	Number of patients	Anatomic site	Histology	Treatment regimen	Outcomes	Comments
Gold et al. [21]	II	55	Esophagus	55/55 adenocarcinoma	Cetuximab 400 mg/m^2^IV × 1, then 250 mg/m^2^ IV weekly	mOS 4 months mPFS 1.8 months	2nd line treatment
							
Ku et al. [22]	II	8	Esophagus/GEJ	7/8 adenocarcinoma (87%) 1/8 squamous cell (13%)	CPT 11 65 mg/m^2^ + Cisplatin 30 mg/m^2^ weekly 2/3 weeks Cetuximab 400 mg/m^2^ × 1, then 250 mg/m^2^ IV weekly	mTTP 4.4 months 1 PR, 2 SD	All patients received prior CPT 11/cisplatin Accrual ongoing
							
Pinto et al. [23]	II	38	34/38 Gastric 4/38 GEJ	38/38 adenocarcinoma	CPT 11 180 mg/m^2^ IV D1 5-FU 400 mg/m^2^ IV bolus D1, 5-FU 600 mg/m^2^ CIVI D1-2, Leucovorin 100 mg/m^2^ IV D1 every 2 weeks × 24 weeks (FOLFIRI) Cetuximab 400 mg/m^2^ × 1, then 250 mg/m^2^ IV weekly	mTTP 8 months median expected survival 16 months 4/34 CR (12%), 11/34 PR (32%), 16/34 SD (47%)	Untreated advanced/ metastatic disease
							
Han et al. [24]	II	38	38/38 gastric	38/38 adenocarcinoma	Oxaliplatin 100 mg/m^2^ IV D1 Leucovorin 100 mg/m^2^ IV D1, 5-FU 1200 mg/m^2^/d CIVI × 46 hours (mFOLFOX6) Cetuximab 400 mg/m^2^ × 1, then 250 mg/m^2^IV weekly	mTTP 5.5 months mOS 9.9 months 19/38 PR (50%), 16/38 SD (42%)	EGF and TGF-alpha levels inversely correlated with response

mOS: median overall survival; mPFS: median progression free survival; PR: partial response; SD: stable disease; GEJ: gastroesophageal junction; TTF: time to failure; TTP: time to progression; CPT 11: irinotecan; EGF: epidermal growth factor; TGF-alpha: transforming growth factor-alpha.
